# Di-μ-thio­cyanato-κ^2^ *N*:*S*;κ^2^ *S*:*N*-bis­({2-morpholino-*N*-[1-(2-pyrid­yl)ethyl­idene]ethanamine-κ^3^ *N*,*N*′,*N*′′}(thio­cyanato-κ*N*)cadmium)

**DOI:** 10.1107/S1600536811002480

**Published:** 2011-01-22

**Authors:** Nura Suleiman Gwaram, Nurul Azimah Ikmal Hisham, Hamid Khaledi, Hapipah Mohd Ali

**Affiliations:** a Department of Chemistry, University of Malaya, 50603 Kuala Lumpur, Malaysia

## Abstract

In the title complex, [Cd_2_(NCS)_4_(C_13_H_19_N_3_O)_2_], the two Cd^II^ ions are bridged by a pair of thio­cyanate *N*:*S*-bridging ligands around an inversion center. One terminal thio­cyanate N atom and one *N*,*N*′,*N*′′-tridentate Schiff base ligand complete a distorted CdN_5_S octa­hedral geometry about each Cd^II^ atom. In the crystal, the Schiff base aromatic rings of adjacent mol­ecules are arranged above each other into infinite chains along the *a* axis with alternate centroid–centroid separations of 3.5299 (13) and 3.7857 (13) Å.

## Related literature

For the structure of the Cu(II) complex with the same Schiff base and thio­cyanate, see: Suleiman Gwaram *et al.* (2011 ▶). For the structures of similar cadmium complexes, see: Banerjee *et al.* (2005 ▶); You *et al.* (2006 ▶).
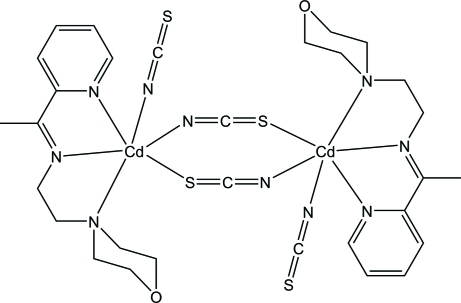


## Experimental

### 

#### Crystal data

[Cd_2_(NCS)_4_(C_13_H_19_N_3_O)_2_]
*M* *_r_* = 923.74
Monoclinic, 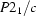

*a* = 7.2934 (2) Å
*b* = 26.4035 (5) Å
*c* = 10.0111 (3) Å
β = 107.853 (3)°
*V* = 1835.02 (9) Å^3^
*Z* = 2
Mo *K*α radiation
μ = 1.43 mm^−1^
*T* = 100 K
0.38 × 0.23 × 0.07 mm

#### Data collection

Bruker APEXII CCD diffractometer
Absorption correction: multi-scan (*SADABS*; Sheldrick, 1996 ▶) *T*_min_ = 0.613, *T*_max_ = 0.907
15344 measured reflections
4008 independent reflections
3638 reflections with *I* > 2σ(*I*)
*R*_int_ = 0.026

#### Refinement

*R*[*F* ^2^ > 2σ(*F* ^2^)] = 0.023
*wR*(*F* ^2^) = 0.047
*S* = 1.10
4008 reflections
218 parameters
2 restraints
H-atom parameters constrained
Δρ_max_ = 0.39 e Å^−3^
Δρ_min_ = −0.45 e Å^−3^

### 

Data collection: *APEX2* (Bruker, 2007 ▶); cell refinement: *SAINT* (Bruker, 2007 ▶); data reduction: *SAINT*; program(s) used to solve structure: *SHELXS97* (Sheldrick, 2008 ▶); program(s) used to refine structure: *SHELXL97* (Sheldrick, 2008 ▶); molecular graphics: *X-SEED* (Barbour, 2001 ▶); software used to prepare material for publication: *SHELXL97* and *publCIF* (Westrip, 2010 ▶).

## Supplementary Material

Crystal structure: contains datablocks I, global. DOI: 10.1107/S1600536811002480/om2400sup1.cif

Structure factors: contains datablocks I. DOI: 10.1107/S1600536811002480/om2400Isup2.hkl

Additional supplementary materials:  crystallographic information; 3D view; checkCIF report

## Figures and Tables

**Fig. 1.**
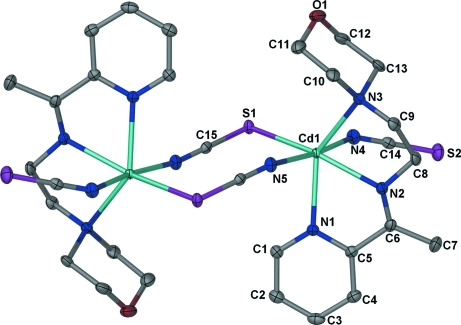
Thermal ellipsoid plot of the title compound at the 50% probability level. Hydrogen atoms have been omitted for clarity. The unlabelled atoms are generated by the symmetry operation (–x + 2, –y, –z + 1).

## supplementary crystallographic information

### Comment

The title compound is a mixed-ligand cadmium(II) complex with thiocyanate and the Schiff base 2-morpholino-*N*-[1-(2-pyridyl)ethylidene]ethanamine. Unlike the mononuclear square-pyramidal structure of the analogous copper(II) complex (Suleiman Gwaram *et al.*, 2011), the present structure represents a dinuclear metal complex with a distorted octahedral geometry around the cadmium atoms. Each two Cd^II^ centers are linked by a pair of thiocyanate *N:S* bridges around an inversion center to form an eight-membered Cd_2_(*µ_2_-*NCS)_2_ ring. The resulting ring has a chair conformation, the displacement of Cd1 out of the (NCS)_2_ plane being 0.635 (2) Å. Within this double bridged dimer, the Cd···Cd distance [5.9380 (3) Å] is similar to those observed in the related complexes (Banerjee *et al.*, 2005; You *et al.*, 2006). The distorted octahedral geometry about the metal cadmium is completed by one terminal thiocyanate N atom and one *N,N',N"*-tridentate Schiff base ligand. In the crystal, the molecules are connected into infinite chains along the *a* axis *via π-π* interactions formed by the Schiff base aromatic ring and its symmetry related counterparts at (-*x* + 1, -*y*, -*z* and –*x* + 2, -*y*, -*z*) with centroid separations of 3.5299 (13) Å and 3.7857 (13) Å respectively.

### Experimental

A mixture of 2-acetylpyridine (0.20 g, 1.65 mmol) and 4-(2-aminoethyl)morpholine (0.21 g, 1.65 mmol) in ethanol (20 ml) was refluxed for 2 hr followed by addition of a solution of cadmium(II) acetate dihydrate (0.44 g, 1.65 mmol) and sodium thiocyanate (0.268 g, 3.30 mmol) in a minimum amount of water. The resulting solution was refluxed for 30 min, then left at room temperature. The crystals of the title complex were obtained in a week.

### Refinement

Hydrogen atoms were placed at calculated positions (C—H 0.95–0.99 Å) and were treated as riding on their parent atoms, with *U*iso(H) set to 1.2–1.5 times *U*eq(C). Additional rigid-bond type restraints (DELU in *SHELXL97*) were placed on the displacement parameters of S1 and C15; S2 and C14.

### Crystal data

**Table:**

[Cd_2_(NCS)_4_(C_13_H_19_N_3_O)_2_]	*F*(000) = 928
*M**_r_* = 923.74	*D*_x_ = 1.672 Mg m^−^^3^
Monoclinic, *P*2_1_/*c*	Mo *K*α radiation, λ = 0.71073 Å
Hall symbol: -P 2ybc	Cell parameters from 7745 reflections
*a* = 7.2934 (2) Å	θ = 2.3–30.5°
*b* = 26.4035 (5) Å	µ = 1.43 mm^−^^1^
*c* = 10.0111 (3) Å	*T* = 100 K
β = 107.853 (3)°	Plate, colourless
*V* = 1835.02 (9) Å^3^	0.38 × 0.23 × 0.07 mm
*Z* = 2	

### Data collection

**Table:**

Bruker APEXII CCD diffractometer	4008 independent reflections
Radiation source: fine-focus sealed tube	3638 reflections with *I* > 2σ(*I*)
graphite	*R*_int_ = 0.026
φ and ω scans	θ_max_ = 27.0°, θ_min_ = 2.3°
Absorption correction: multi-scan (*SADABS*; Sheldrick, 1996)	*h* = −9→9
*T*_min_ = 0.613, *T*_max_ = 0.907	*k* = −33→33
15344 measured reflections	*l* = −12→12

### Refinement

**Table:**

Refinement on *F*^2^	Primary atom site location: structure-invariant direct methods
Least-squares matrix: full	Secondary atom site location: difference Fourier map
*R*[*F*^2^ > 2σ(*F*^2^)] = 0.023	Hydrogen site location: inferred from neighbouring sites
*wR*(*F*^2^) = 0.047	H-atom parameters constrained
*S* = 1.10	*w* = 1/[σ^2^(*F*_o_^2^) + (0.0141*P*)^2^ + 1.4854*P*] where *P* = (*F*_o_^2^ + 2*F*_c_^2^)/3
4008 reflections	(Δ/σ)_max_ = 0.001
218 parameters	Δρ_max_ = 0.39 e Å^−^^3^
2 restraints	Δρ_min_ = −0.45 e Å^−^^3^

### Special details

**Table:**

Geometry. All e.s.d.'s (except the e.s.d. in the dihedral angle between two l.s. planes) are estimated using the full covariance matrix. The cell e.s.d.'s are taken into account individually in the estimation of e.s.d.'s in distances, angles and torsion angles; correlations between e.s.d.'s in cell parameters are only used when they are defined by crystal symmetry. An approximate (isotropic) treatment of cell e.s.d.'s is used for estimating e.s.d.'s involving l.s. planes.
Refinement. Refinement of *F*^2^ against ALL reflections. The weighted *R*-factor *wR* and goodness of fit *S* are based on *F*^2^, conventional *R*-factors *R* are based on *F*, with *F* set to zero for negative *F*^2^. The threshold expression of *F*^2^ > σ(*F*^2^) is used only for calculating *R*-factors(gt) *etc*. and is not relevant to the choice of reflections for refinement. *R*-factors based on *F*^2^ are statistically about twice as large as those based on *F*, and *R*- factors based on ALL data will be even larger.

### Fractional atomic coordinates and isotropic or equivalent isotropic displacement parameters (Å^2^)

**Table:**

	*x*	*y*	*z*	*U*_iso_*/*U*_eq_	
Cd1	0.88459 (2)	0.089861 (5)	0.314896 (15)	0.01292 (5)	
S1	0.67607 (7)	0.05268 (2)	0.46956 (6)	0.01758 (11)	
S2	0.45365 (9)	0.20002 (2)	−0.00994 (6)	0.02685 (13)	
O1	1.0113 (3)	0.19368 (6)	0.65208 (18)	0.0309 (4)	
N1	0.7974 (2)	0.02944 (6)	0.12874 (18)	0.0153 (4)	
N2	0.9534 (2)	0.11963 (7)	0.11679 (18)	0.0166 (4)	
N3	1.0711 (3)	0.16656 (7)	0.38989 (19)	0.0166 (4)	
N4	0.6008 (3)	0.13692 (7)	0.2228 (2)	0.0214 (4)	
N5	1.1395 (3)	0.03924 (7)	0.42969 (19)	0.0194 (4)	
C1	0.7455 (3)	−0.01826 (8)	0.1403 (2)	0.0181 (4)	
H1	0.7435	−0.0300	0.2296	0.022*	
C2	0.6942 (3)	−0.05172 (9)	0.0281 (2)	0.0217 (5)	
H2	0.6593	−0.0857	0.0402	0.026*	
C3	0.6952 (3)	−0.03430 (9)	−0.1020 (2)	0.0226 (5)	
H3	0.6596	−0.0561	−0.1812	0.027*	
C4	0.7486 (3)	0.01527 (9)	−0.1153 (2)	0.0203 (5)	
H4	0.7495	0.0279	−0.2040	0.024*	
C5	0.8011 (3)	0.04641 (8)	0.0022 (2)	0.0163 (4)	
C6	0.8705 (3)	0.09967 (8)	−0.0016 (2)	0.0183 (4)	
C7	0.8396 (4)	0.12483 (10)	−0.1409 (2)	0.0303 (6)	
H7A	0.9115	0.1567	−0.1277	0.045*	
H7B	0.7020	0.1317	−0.1840	0.045*	
H7C	0.8849	0.1025	−0.2023	0.045*	
C8	1.0294 (3)	0.17102 (8)	0.1330 (2)	0.0212 (5)	
H8A	0.9225	0.1957	0.1163	0.025*	
H8B	1.0991	0.1775	0.0640	0.025*	
C9	1.1654 (3)	0.17704 (8)	0.2811 (2)	0.0203 (5)	
H9A	1.2757	0.1537	0.2943	0.024*	
H9B	1.2165	0.2120	0.2932	0.024*	
C10	1.2234 (3)	0.16048 (9)	0.5272 (2)	0.0228 (5)	
H10A	1.3057	0.1911	0.5475	0.027*	
H10B	1.3056	0.1311	0.5230	0.027*	
C11	1.1333 (4)	0.15259 (9)	0.6422 (2)	0.0285 (5)	
H11A	1.0571	0.1209	0.6241	0.034*	
H11B	1.2364	0.1488	0.7328	0.034*	
C12	0.8634 (4)	0.19928 (9)	0.5217 (3)	0.0266 (5)	
H12A	0.7776	0.2276	0.5287	0.032*	
H12B	0.7852	0.1680	0.5009	0.032*	
C13	0.9472 (3)	0.20975 (8)	0.4035 (2)	0.0207 (5)	
H13A	0.8418	0.2144	0.3145	0.025*	
H13B	1.0243	0.2413	0.4233	0.025*	
C14	0.5395 (3)	0.16332 (8)	0.1265 (2)	0.0164 (4)	
C15	0.7869 (3)	−0.00104 (8)	0.5288 (2)	0.0142 (4)	

### Atomic displacement parameters (Å^2^)

**Table:**

	*U*^11^	*U*^22^	*U*^33^	*U*^12^	*U*^13^	*U*^23^
Cd1	0.01395 (7)	0.01189 (8)	0.01358 (8)	0.00068 (6)	0.00518 (5)	0.00135 (6)
S1	0.0165 (2)	0.0166 (3)	0.0221 (3)	0.0035 (2)	0.0095 (2)	0.0053 (2)
S2	0.0255 (3)	0.0263 (3)	0.0251 (3)	0.0015 (2)	0.0024 (2)	0.0085 (2)
O1	0.0435 (11)	0.0281 (9)	0.0257 (9)	−0.0086 (8)	0.0172 (8)	−0.0098 (7)
N1	0.0132 (8)	0.0162 (9)	0.0167 (9)	0.0012 (7)	0.0048 (7)	−0.0004 (7)
N2	0.0159 (9)	0.0168 (9)	0.0191 (9)	0.0013 (7)	0.0085 (7)	0.0014 (7)
N3	0.0178 (9)	0.0150 (9)	0.0192 (9)	−0.0010 (7)	0.0088 (7)	−0.0012 (7)
N4	0.0170 (9)	0.0219 (10)	0.0260 (10)	0.0029 (8)	0.0074 (8)	0.0023 (8)
N5	0.0156 (9)	0.0207 (10)	0.0229 (10)	−0.0002 (8)	0.0072 (8)	0.0036 (8)
C1	0.0144 (10)	0.0195 (11)	0.0204 (11)	0.0000 (8)	0.0054 (9)	0.0001 (9)
C2	0.0157 (10)	0.0200 (12)	0.0277 (12)	0.0003 (9)	0.0044 (9)	−0.0036 (9)
C3	0.0156 (10)	0.0259 (12)	0.0231 (12)	0.0032 (9)	0.0014 (9)	−0.0089 (9)
C4	0.0168 (10)	0.0269 (12)	0.0165 (11)	0.0041 (9)	0.0044 (9)	−0.0009 (9)
C5	0.0127 (9)	0.0198 (11)	0.0169 (10)	0.0034 (8)	0.0053 (8)	−0.0003 (8)
C6	0.0196 (10)	0.0200 (12)	0.0182 (11)	0.0057 (9)	0.0102 (9)	0.0027 (8)
C7	0.0436 (15)	0.0288 (13)	0.0213 (12)	0.0030 (11)	0.0140 (11)	0.0061 (10)
C8	0.0271 (12)	0.0170 (11)	0.0243 (12)	−0.0023 (9)	0.0150 (10)	0.0032 (9)
C9	0.0198 (11)	0.0184 (11)	0.0268 (12)	−0.0040 (9)	0.0133 (10)	−0.0006 (9)
C10	0.0233 (11)	0.0201 (12)	0.0222 (12)	−0.0053 (9)	0.0030 (9)	−0.0018 (9)
C11	0.0356 (14)	0.0290 (13)	0.0191 (12)	−0.0071 (11)	0.0054 (10)	−0.0028 (10)
C12	0.0320 (13)	0.0189 (12)	0.0359 (14)	−0.0058 (10)	0.0207 (11)	−0.0097 (10)
C13	0.0252 (12)	0.0117 (10)	0.0279 (12)	−0.0011 (9)	0.0122 (10)	−0.0026 (9)
C14	0.0122 (9)	0.0157 (10)	0.0214 (10)	0.0007 (8)	0.0053 (8)	−0.0012 (7)
C15	0.0115 (9)	0.0174 (9)	0.0150 (10)	−0.0011 (7)	0.0060 (8)	0.0015 (8)

### Geometric parameters (Å, °)

**Table:**

Cd1—N5	2.2922 (18)	C3—H3	0.9500
Cd1—N2	2.3267 (17)	C4—C5	1.389 (3)
Cd1—N4	2.3469 (18)	C4—H4	0.9500
Cd1—N1	2.3866 (17)	C5—C6	1.499 (3)
Cd1—N3	2.4269 (17)	C6—C7	1.498 (3)
Cd1—S1	2.6679 (5)	C7—H7A	0.9800
S1—C15	1.650 (2)	C7—H7B	0.9800
S2—C14	1.635 (2)	C7—H7C	0.9800
O1—C12	1.423 (3)	C8—C9	1.518 (3)
O1—C11	1.426 (3)	C8—H8A	0.9900
N1—C1	1.330 (3)	C8—H8B	0.9900
N1—C5	1.352 (3)	C9—H9A	0.9900
N2—C6	1.268 (3)	C9—H9B	0.9900
N2—C8	1.456 (3)	C10—C11	1.505 (3)
N3—C9	1.482 (3)	C10—H10A	0.9900
N3—C10	1.487 (3)	C10—H10B	0.9900
N3—C13	1.487 (3)	C11—H11A	0.9900
N4—C14	1.162 (3)	C11—H11B	0.9900
N5—C15^i^	1.158 (3)	C12—C13	1.514 (3)
C1—C2	1.388 (3)	C12—H12A	0.9900
C1—H1	0.9500	C12—H12B	0.9900
C2—C3	1.383 (3)	C13—H13A	0.9900
C2—H2	0.9500	C13—H13B	0.9900
C3—C4	1.384 (3)	C15—N5^i^	1.158 (3)
			
N5—Cd1—N2	105.70 (6)	N2—C6—C5	115.71 (19)
N5—Cd1—N4	171.05 (6)	C7—C6—C5	118.9 (2)
N2—Cd1—N4	83.16 (6)	C6—C7—H7A	109.5
N5—Cd1—N1	89.01 (6)	C6—C7—H7B	109.5
N2—Cd1—N1	68.63 (6)	H7A—C7—H7B	109.5
N4—Cd1—N1	93.18 (6)	C6—C7—H7C	109.5
N5—Cd1—N3	92.28 (6)	H7A—C7—H7C	109.5
N2—Cd1—N3	74.62 (6)	H7B—C7—H7C	109.5
N4—Cd1—N3	91.32 (6)	N2—C8—C9	108.61 (18)
N1—Cd1—N3	142.12 (6)	N2—C8—H8A	110.0
N5—Cd1—S1	90.78 (5)	C9—C8—H8A	110.0
N2—Cd1—S1	158.33 (4)	N2—C8—H8B	110.0
N4—Cd1—S1	80.32 (5)	C9—C8—H8B	110.0
N1—Cd1—S1	98.30 (4)	H8A—C8—H8B	108.3
N3—Cd1—S1	119.52 (4)	N3—C9—C8	112.74 (18)
C15—S1—Cd1	102.77 (7)	N3—C9—H9A	109.0
C12—O1—C11	109.23 (17)	C8—C9—H9A	109.0
C1—N1—C5	118.92 (18)	N3—C9—H9B	109.0
C1—N1—Cd1	125.41 (14)	C8—C9—H9B	109.0
C5—N1—Cd1	115.67 (13)	H9A—C9—H9B	107.8
C6—N2—C8	123.20 (19)	N3—C10—C11	110.18 (19)
C6—N2—Cd1	119.39 (14)	N3—C10—H10A	109.6
C8—N2—Cd1	113.20 (13)	C11—C10—H10A	109.6
C9—N3—C10	108.44 (17)	N3—C10—H10B	109.6
C9—N3—C13	110.88 (17)	C11—C10—H10B	109.6
C10—N3—C13	107.49 (17)	H10A—C10—H10B	108.1
C9—N3—Cd1	105.72 (12)	O1—C11—C10	112.1 (2)
C10—N3—Cd1	112.63 (13)	O1—C11—H11A	109.2
C13—N3—Cd1	111.67 (13)	C10—C11—H11A	109.2
C14—N4—Cd1	135.42 (16)	O1—C11—H11B	109.2
C15^i^—N5—Cd1	154.57 (17)	C10—C11—H11B	109.2
N1—C1—C2	123.1 (2)	H11A—C11—H11B	107.9
N1—C1—H1	118.5	O1—C12—C13	111.19 (19)
C2—C1—H1	118.5	O1—C12—H12A	109.4
C3—C2—C1	118.2 (2)	C13—C12—H12A	109.4
C3—C2—H2	120.9	O1—C12—H12B	109.4
C1—C2—H2	120.9	C13—C12—H12B	109.4
C2—C3—C4	119.2 (2)	H12A—C12—H12B	108.0
C2—C3—H3	120.4	N3—C13—C12	109.51 (18)
C4—C3—H3	120.4	N3—C13—H13A	109.8
C3—C4—C5	119.4 (2)	C12—C13—H13A	109.8
C3—C4—H4	120.3	N3—C13—H13B	109.8
C5—C4—H4	120.3	C12—C13—H13B	109.8
N1—C5—C4	121.2 (2)	H13A—C13—H13B	108.2
N1—C5—C6	116.03 (18)	N4—C14—S2	179.4 (2)
C4—C5—C6	122.76 (19)	N5^i^—C15—S1	178.34 (19)
N2—C6—C7	125.3 (2)		

Symmetry codes: (i) −*x*+2, −*y*, −*z*+1.
